# Metallothioneins, Unconventional Proteins from Unconventional Animals: A Long Journey from Nematodes to Mammals ^†^

**DOI:** 10.3390/biom4020435

**Published:** 2014-04-22

**Authors:** Gloria Isani, Emilio Carpenè

**Affiliations:** Department of Veterinary Medical Sciences, Alma Mater Studiorum-University of Bologna, via Tolara di sopra, 50, Ozzano Emilia, Bologna 40064, Italy; E-Mail: emilio.carpene@unibo.it

**Keywords:** metallothioneins, isoforms, metals, nematodes, arthropods, mollusks, annelids echinoderms, vertebrates

## Abstract

Metallothioneins (MTs) are ubiquitous low molecular weight cysteine-rich proteins characterized by high affinity for d10 electron configuration metals, including essential (Zn and Cu) and non-essential (Cd and Hg) trace elements. The biological role of these ancient and well-conserved multifunctional proteins has been debated since MTs were first discovered in 1957. Their main hypothesized functions are: (1) homeostasis of Zn and Cu; (2) detoxification of Cd, and Hg; and (3) free radical scavenging. This review will focus on MTs in unconventional animals, those not traditionally studied in veterinary medicine but of increasing interest in this field of research. Living in different environments, these animals represent an incredible source of physiological and biochemical adaptations still partly unexplored. The study of metal-MT interactions is of great interest for clinicians and researchers working in veterinary medicine, food quality and endangered species conservation.

## 1. Introduction

Metallothionein (MT) was first isolated in 1957 from horse kidney [1] and subsequently characterized [2], showing that different isoforms are present depending on the species. MTs are intracellular low molecular weight cysteine-rich proteins characterized by high affinity for d10 electron configuration metals, including essential (Zn and Cu) and non-essential (Cd and Hg) trace elements. MTs are ubiquitous and have been isolated in a wide variety of organisms from bacteria to humans playing a key role in metal metabolism. A PubMed search, using “metallothionein” as key word, yielded more than 10,300 publications since 1960, with an average of around 400 per year in the last decade, on myriad topics from physical chemistry to applied medicine and environmental monitoring. By contrast, less information is available on MTs in unconventional animals. This review considers “unconventional” those species not traditionally studied in human and veterinary medicine, such as snails, octopuses, snakes, turtles, birds and dolphins. Living in different environments, all these animals represent an incredible source of physiological and biochemical adaptations and are of increasing interest in many fields of research, including veterinary and human medicine.

## 2. Methods of MT Quantification

**Figure 1:**
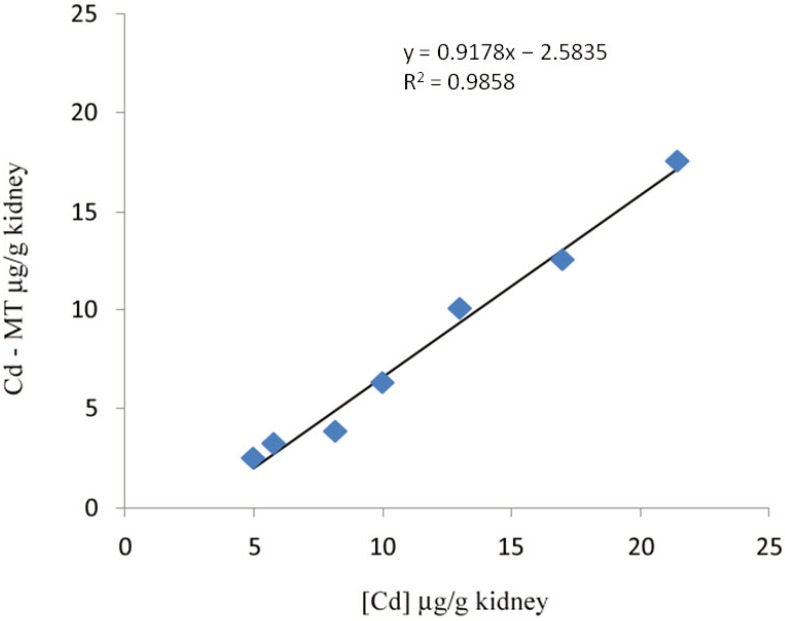
Linear regression between Cd concentration and CdMT in kidney of Eurasian woodcock. Adapted from Carpenè *et al.* [9].

MT quantification methods are based either on direct protein measurement or indirect determination. Additional methods rely on monitoring MT-mRNA by real time PCR, a technique able to distinguish different isoform transcripts, but these do not always match with the corresponding thioneins [3]. There is currently no simple MT quantification method, though accurate measurement of the protein is mandatory to understand both its functions and biomarker potential. Recent advances in MT speciation research resulted in a variety of promising techniques, including HPLC-ICP-MS, HPLC-ESI-MS and RP-HPLC coupled to fluorescence detection. However, in spite of the sensitivity and accuracy of these new methods, the analysis of biological samples with these hyphenated techniques requires expensive equipment and well-trained technicians [4]. Size-exclusion liquid chromatography associated with atomic absorption spectrometry for metal analysis is generally used for MT isolation and quantification. Though this method may underestimate MT concentration, it has the great advantage of isolating MT under mild physiological conditions, keeping the protein as intact as possible still binding the metals that were actually bound *in vivo* [5]. Other common methods based on sulphydryl quantification are easy to perform but lack specificity [6]. Ryvolova *et al.* [7] recently published an exhaustive review on analytical methods for MT detection including Raman spectroscopy, considered a promising new technique [8]. On the other hand, omitting the complex hyphenated techniques mentioned above, MT can be indirectly quantified in a tissue when a significant correlation is found between metal tissue concentration and MT. An example is reported for CdMT in kidney of Eurasian woodcock (*Scolopax rusticola*) (Figure 1), where the CdMT can be simply calculated from the metal concentrations in tissue [9].

Difficulties in MT isolation are also related to the kind of the sequestered metal, e.g., CuMT readily undergoes oxidation and its purification must be carried out under anaerobic and reducing conditions to avoid polymerization [10]. In conclusion, notwithstanding the different analytical methods applied in the fields of environmental sciences, biochemistry, clinical biochemistry and pathology, a simple, specific, accurate and sensitive method for MT quantification remains a stimulating challenge.

## 3. Molecular Characteristics and Isoforms

**Figure 2:**
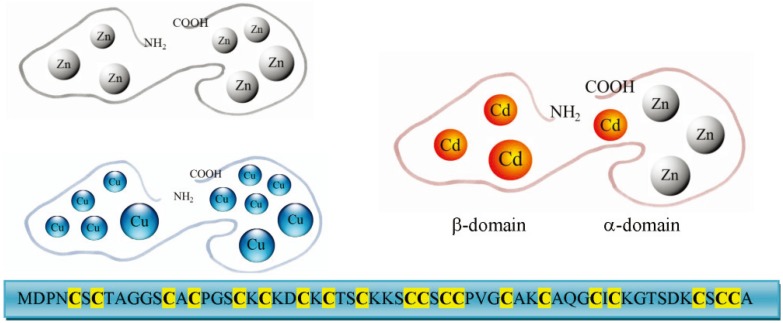
Schematic representation of metallothionein (MT) structures that show the two domains in a simplified geometry. Depending on the bound metal ions different stoichiometries are shown: Zn_7_MT, Cu_11_MT and Cd_4_Zn_3_MT. The sequence of the MT from the dolphin, *Tursiops truncatus*, is also reported.

The first amino acid sequences of mammalian MTs obtained in the Seventies revealed that these proteins contained 61–62 amino acids, of which 20 were highly conserved cysteines characterized by the absence of aromatic amino acids. Thioneins do not present secondary structures and adopt their specific three-dimensional structure after the binding of metal ions. Determination of this structure disclosed trinuclear and tetranuclear thiolate clusters with divalent metal ions [11]. Each metal is sequestered by four cysteines in a tetrahedral geometry, with an assumed Cd-S distance of 2.60 Å. These complexes are characterized by high thermodynamic stability whereas the metals within the cluster are in a dynamic state, with continuous redistribution of ions favoured by the nonrigidity of MTs [12]. MT can bind monovalent and bivalent metal ions with different stoichiometries (Figure 2). By coincidence, unconventional MT structures are present in unconventional animals and will be discussed below.

Typically, mammals present four main isoforms from MT-1 to MT-4, with the most represented, MT-1 and MT-2, expressed in many tissues, particularly in kidney and liver. These MTs are conserved proteins with an important role in metal homeostasis and detoxification. By contrast, MT-3 shows a characteristic six amino acid insertion in the α-domain and an additional threonine in the β-domain. This isoform, formerly considered specific for the brain, has also been found in other tissues. The latest isoform discovered is MT-4, which displays the highest diversity compared to the other mammalian MTs and is expressed in squamous epithelia. The highest isoform complexity has been reported in humans, where MTs are encoded by a family of at least ten different genes located on chromosome 16q13. The superfamily of conventional MTs has probably evolved from an ancestral gene, through gene duplication and loss, followed by subsequent evolutionary divergence leading to a complex and heterogeneous pattern, even if a convergent evolution yielding “MT-like proteins” cannot be excluded.

## 4. Functions

**Figure 3:**
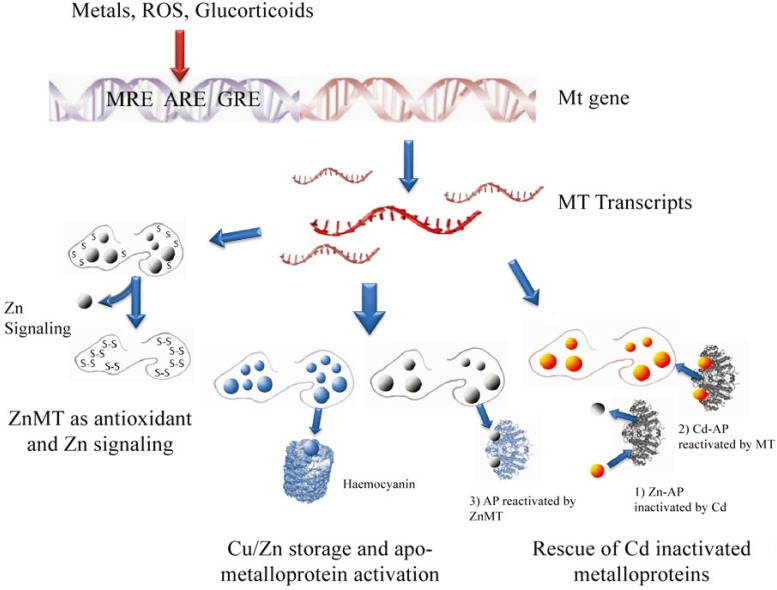
In the present figure are reported the main hypothesized functions of MT (bottom) and a simple scheme of MT gene regulation (top). AP: Alkaline Phosphatase; MRE: metal response element; ARE: antioxidant response element; GRE: glucocorticoid response elements.

Despite over half a century of research, the exact functions of MTs are still under debate [13,14,15]. Due to the high kinetic mobility of metals within metal clusters, MTs are involved in trace metal exchange (binding/release). The main hypothesized functions (Figure 3) are: (1) homeostasis of the essential trace metals Zn and Cu; (2) detoxification of the non-essential metals Cd and Hg; (3) donation of essential metals to apo metalloproteins; (4) protection against oxidative damage; (5) free radical scavenger as thionein a [16,17]; last but not least, MT could be a multifunctional protein. MT functions can be investigated at different levels of its molecular life spanning from the gene structure and transcription to the thionein degradation. The genomic organization of the MT genes is quite conserved: the typical structure of the coding region includes three exons and two introns. Transcription of metallothionein is regulated in response to different stimuli, particularly heavy metals, oxidative stress and glucocorticoids [13]; accordingly, metallothionein promoter region from most organisms contains specific response sequences termed metal response elements (MREs), antioxidant response elements (AREs) and glucocorticoid response elements (GREs). In this respect, special attention is paid to the metal transcription factor MTF-1 [18], that in turn is a metallo-protein belonging to the zinc-finger family, first discovered in the unconventional amphibian *Xenopus laevis* [19].

## 5. Unconventional Animals

**Figure 4:**
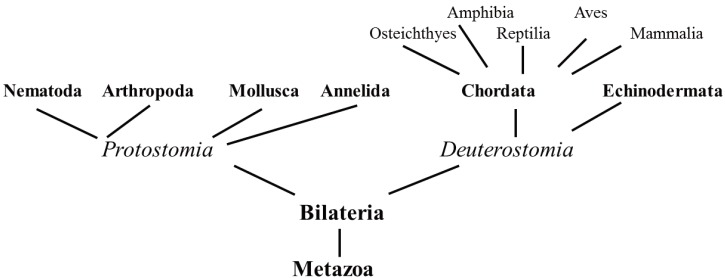
Simplified scheme highlighting the taxonomic groups considered in this review.

In the past, veterinary medicine was mainly interested in a few species of vertebrate animals of economic importance, large animals for work and food production, dogs and cats as companion animals and their respective pathogens. Nowadays, crustaceans, mollusks and several species of vertebrates are increasingly exploited as pets or as food. As a consequence, veterinary sciences have recently focused on myriad unconventional species spread along the phylogenetic tree. The animal evolutionary tree is still under debate and molecular techniques are often used to explore a large number of sequences, including those of MTs, to analyze the relationships among different species [20,21]. Our review adopted an evolutionary approach, presenting data on selected species from the main Protostomia phyla, Nematoda, Annelida, Arthropoda and Mollusca. From the Deuterostomia we have chosen examples from Echinodermata and Chordata phyla, the latter focused on Vertebrate classes of Osteichthyes, Amphibia, Reptilia, Aves and Mammalia (Figure 4). Last but not least, many of the species we investigated for our metallomic studies belong to these taxonomic groups.

### 5.1. Protostomia

Protostomia are a heterogeneous clade of invertebrates characterized by bilateral symmetry and the mouth developing directly from the blastopore. This clade includes large phyla like Arthropoda and Mollusca presenting a huge number of morpho-physiological and biochemical adaptations, including a multiplicity of MT isoforms.

#### 5.1.1. Nematoda

Nematoda, one of the richest phyla after the Arthropoda, comprises at least 80,000 different species, including many free-living species and a large number of parasitic species of vertebrates. However, GenBank contains the MT sequences of only four nematode species, *Trichinella spiralis*, *Caenorhabditis elegans*, *Caenorhabditis remanei*, and *Caenorhabditis briggsae*, and in-depth studies have only been carried out on the model molecular biology organism *C. elegans*. The genome of this nematode encodes two multifunctional metal-binding MTs, CeMT-1 and CeMT-2 [22], isolated for the first time after ion-exchange chromatography [23] and further characterized. CeMT-1, 75 residues long, presents four histidines, whereas CeMT-2, 63 residues long, has only one histidine, an amino acid essential for the coordination performance of the two isoforms [24] (Table 1). Differences between *C. elegans* and mammalian MTs are evident, e.g., the position of the cysteine residues differs and the *N*-terminal amino acids are alanine and valine in CeMT-I and -II respectively, whereas they are methionine in mammalian MTs [25]. In *C. elegans* both isoforms contribute to Cd detoxification, but they are not essential for life and the compensatory action of phytochelatins can protect against Cd toxicity [26]; moreover, *C. elegans* lacks MREs in the promoter region of MT gene [22].

#### 5.1.2. Annelida

Annelida are a large phylum comprising more than 2000 species, which have colonized either aquatic or terrestrial environments. One of the most widely studied groups is the Oligochaeta, a subclass playing a key role in the maintenance of soil structure and fertility. Since 1984, these organisms have been exploited as bioindicators in the ecotoxicological assessment of environmental pollution [27]. *Eisenia fetida* and *Lumbricus rubellus* are the most widely studied species, the first evidence of MT in *E. fetida* being reported by Suzuki *et al.* [28]. Gruber *et al.* [29] subsequently obtained a cDNA coding for a putative MT of 80 amino acids, but the *in vivo* experiment disclosed only a shorter MT sequence of 41 residues. Two hypotheses were proposed: firstly, the formation of small MT peptides due to enzymatic cleavage of the intact thionein during the isolation procedure; secondly, a deliberate post-translational modification to yield a functional one-domain MT [29]. In addition to MT, when exposed to Cd *E. fetida* also produces phytochelatin synthase [30] as also reported in *C. elegans*. Unlike *E. fetida*, two MT isoforms, displaying an identity of 74.7% and a similarity of 91.1%, have been isolated in *L. rubellus*. All cysteine residues are conserved between the two isoforms, but interestingly there is a distinctive two amino acid deletion/insertion [31]. Whereas Oligochaeta are used to monitor terrestrial heavy metal pollution, Polychaeta could be useful bioindicators for assessing coastal marine pollution, but few studies have investigated MTs in these invertebrates [32]. A MT containing 68 aminoacids was found in the polychaete *Perinereis nuntia*; the 17 cysteine residues were distributed along the entire sequence with five positions adjacent to lysine [20].

**Table 1:** Examples of different MT primary structure from unconventional species. In the first line human (*Homo sapiens*) MT-2A is reported for comparison. Cysteines are evidenced in yellow, lysines in green and histidine in red. Histidine has been evidenced because of its possible involvement in metal binding. Cys: cysteine; AA: aminoacid. The sequences are referred to dolphin (*Tursiops truncatus*), cormorant (*Phalacrocorax carbo*), lizard (*Podarcis sicula*), newt (*Triturus carnifex*), goldfish (*Carassius auratus*), sea urchin (*Strongylocentrotus purpuratus*), mussel (*Mytilus edulis*), arcid clam (*Scapharca inaequivalvis*), snail (*Helix pomatia*), blue crab (*Callinectes sapidus*), common fruit fly (*Drosophila melanogaster*), polychaete (*Perinereis nuntia*), earthworm (*Lumbricus rubellus*) and nematode (*Caernorabditis elegans*). All the sequences were obtained from GenBank. * The sequence of *S. inaequivalvis* MT is not complete.

Species	Name	Sequence	Cys	AA
*H. sapiens*	MT-SA	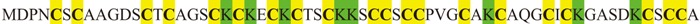	20	61
**Vertebrates**				
*T. truncates*	MT-2A	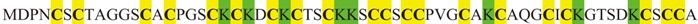	20	61
*P. carbo*	MT-2	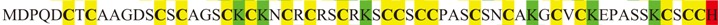	20	63
*P. sicula*	MT	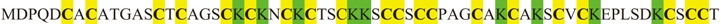	20	63
*T. carnifex*	MT	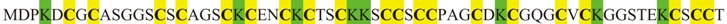	20	63
*C. auratus*	MT	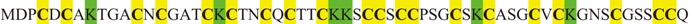	20	60
**Invertebrates**				
*S. purpuratus*	SpMTA	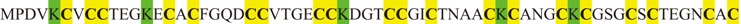	20	64
*M. edulis*	MT10Ib	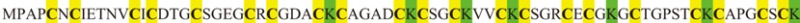	21	73
*S. inaequivalvis*	MT	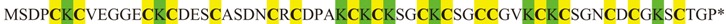	18	62
*H. pomatia*	CuMT	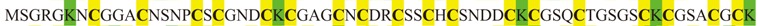	18	65
*C. sapidus*	CuMT-2	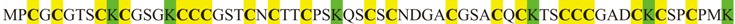	21	64
*D. melanogaster*	MtnE	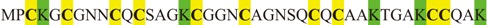	10	41
*P. nuntia*	MT	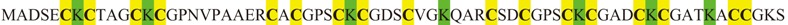	17	68
*L. rubellus*	MT-1	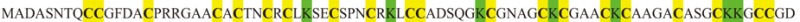	20	79
*C. elegans*	CeMT-1	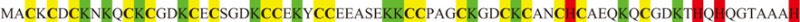	19	75

#### 5.1.3. Arthropoda

Arthropoda are the largest animal phylum, comprising at least one million species, living in either terrestrial or aquatic environments. As a consequence, the classification is rather complex and includes different subphyla and several classes. All arthropods are characterized by an exoskeleton that can molt several times throughout life. Our review chose a few important species belonging to the subphylum of Crustacea and the classes of Insecta. In veterinary medicine, crustaceans are important mostly in the field of aquaculture because they contain some edible species of crabs and shrimps and other species as fish parasites. Evidence of crustacean MTs was first reported in *Cancer pagurus* by Overnell and Trewhella [33] and later in *Scylla serrata* by Otvos *et al.* [34]. These MTs contain 57 to 58 amino acids and 18 cysteine residues, resulting in the binding of six metal atoms per molecule. Lobster MT also contains 18 cysteines and binds six bivalent metal ions, sequestered in two β-clusters [35]. Differently, a copper-specific CuMT-2 has been isolated in *Callinectes sapidus*. This MT-2 is 64 amino acids long, contains 21 cysteines and is characterized by two Cys-Cys-Cys triplets, each domain containing one triplet. In terms of MT-2’s metal-binding capacity, the fully Zn/Cd metallated thionein could present up to eight metal ions and up to 12 for Cu(I) [36]. Crustaceans present a particular Cu(I) oxygen carrier protein, haemocyanin, and should be characterized by specific homeostatic mechanisms. For example, MT could be a potential donor of Cu(I) to apo-haemocyanin [10]. *Daphnia pulex*, an important model organism in ecotoxicology, presents four MT isoforms: DpMT-1 and DpMT-3 responsive to cadmium stress, and DpMT-2 and DpMT-4 responsive primarily to copper stress [37]. The fruit fly *Drosophila melanogaster* is one of the most studied insects in the fields of genetics and molecular biology and not surprisingly many studies on MT has been carried out in this species. It has been found that *D. melanogaster* genome harbors a family of at least five expressed MT genes, codifying respectively for five MT isoforms MtnA-E [38]. The analysis of fruit fly *interactome* revealed that the Zn-loaded MtnA and MtnB interacted with peroxiredoxin, opening new perspectives on the role of MTs in the redox network [39].

#### 5.1.4. Mollusca

**Figure 5:**
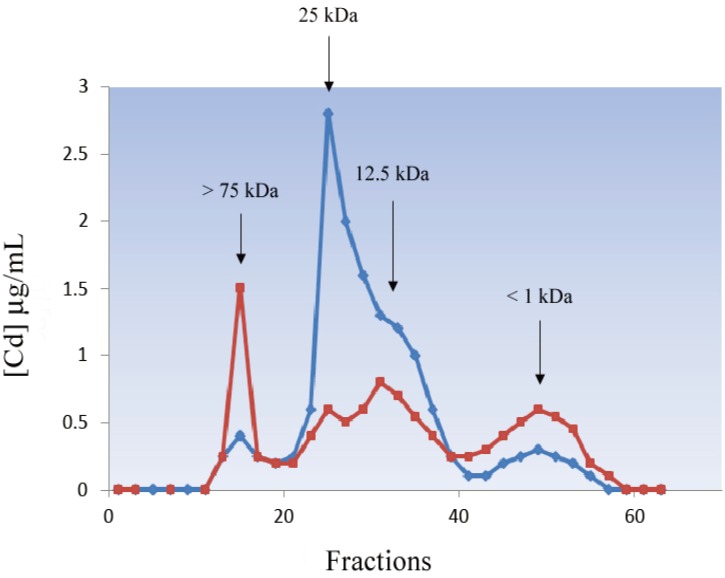
Cd profiles from *Mytilus edulis* hepatopancreas cytosol after gel filtration chromatography on Sephadex G-75 performed immediately after extraction (**blue** line) and after 3 days of storage at −20 °C (**red** line).

The phylum of Mollusca, one of the largest and most diverse monophyletic taxon of Invertebrates, includes animals as different as chitons (sea slug), aplysias, bivalves, snails and octopuses, living mainly in the marine environment, but with some terrestrial and fresh water species. The interest of veterinary medicine in mollusks extends to several fields: (a) aquarium pets, e.g., *Aplysia* spp*.*; (b) model species for the study of animal behavior, e.g., *Octopus*; (c) accumulation of biotoxins or production of biotoxins studied as pharmaceuticals, e.g., *Conidae*; (d) indicators of aquatic and terrestrial pollution, e.g., *Mytilus* spp*.* and *Helix pomatia*; and (e) food production, e.g., mussels, oysters and cephalopods. Bivalvia and Gastropoda, namely mussels and snails have been the most widely studied mollusks due to their availability, easy collection and maintenance in captivity as they are cultivated in large amounts all over the world for human consumption. Due to their colonization of different environments, mollusks show a wide range of physiological and biochemical adaptations to sessile and pelagic life in the aquatic environment. Following the suggestion of Goldberg [40], myriads of studies have utilized mussels to study the effect of metal pollution in different environmental conditions [41,42,43]. Moreover, terrestrial snails experienced the transition from marine to terrestrial conditions and evolved specific adaptations for air breathing, aestivation and hibernation. Several species have adopted specific respiratory proteins for oxygen transport based on free-circulating giant haemocyanins which are not included in cells; differently, the Arcid clam, *Scapharca inaequivalvis*, presents dimeric and tetrameric hemoglobins included in nucleated erythrocytes [44]. Presumably, as a consequence of this huge molecular variability, molluskan MTs also present unconventional adaptations. The first evidence of metal-binding low molecular weight proteins in mollusks was reported in the gastropod *Patella vulgata* [45], in the bivalves *Mytilus edulis* [46], *Mytilus galloprovincialis* [47], *Crassostrea virginica* [48] and in the chiton *Cryptochiton stelleri* [49]. The evolutionary diversity of Mollusca is reflected in the primary structure of MTs and their biophysical properties [14]. Two main isoforms of different molecular weight were isolated by gel filtration on Sephadex G75 in *M. edulis* [50] and *M. galloprovincialis* [51]. These MTs, subsequently named MT-10 and MT-20, contain 72 (21 cysteines) and 71 amino acids (23 cysteines respectively) [52]. The additional cysteine in MT-20 can participate in an intermolecular bridge between the two monomeric subunits. Moreover, these two isoforms differ in stability when stored at −20 °C for 3 days, with MT-10 being more stable (Figure 5). MT-10 and MT-20 originated from an ancestral gene duplication occurring before the separation of the two mussel species. This first duplication event was followed by a number of duplications, within both MT-10 and MT-20, revealing a complex evolutionary pattern of the MT genes and the related thioneins within Mytilidae [52,53,54]. An additional short MT-10 gene has been reported in *M. edulis* and *M. galloprovincialis* [53,55]. MT-10 is expressed constitutively, whereas MT-20 is present at a very low concentration in physiological conditions, though the regulation mechanisms are still poorly understood. Northern hybridization of the MT-mRNA concentration disclosed a rapid response of the MT1-10 gene after Zn exposure, while MT-20 was specifically up-regulated by Cd [56]. We have widely studied metal pollution in the Adriatic Sea using the arcid clam *S. inaequivalvis* as an animal model [57,58,59]. The MT of this unconventional mollusk presents an aminoacid sequence with a high glycine content and a higher number of lysines (13.3%) than in other mollusks [60]. In addition to the two main MT isoforms [61], *Crassostrea gigas* presents another atypical MT gene, *CgMT2*, characterized by an exon duplication, and this also seems to be an active gene [62,63].

The MTs of pulmonate snails provide a paradigm of gastropod MTs. *Helix pomatia* presents different metal-specific isogene families, which show two main isoforms with 65 amino acids and 18 cysteines at conserved positions: a CuMT binding 12 Cu atoms per molecule and involved in Cu homeostasis, and a second Cd-specific isoform binding six divalent metal atoms per molecule specialized in Cd sequestering and detoxification [64,65,66]. Besides these two metal-specific MTs, a third non-specific isoform binding Cu^+^, Cd^2+^ and Zn^2+^ is normally expressed only at very low concentrations. All these isoforms have similar molecular size and possess cysteines at invariant positions, but differ widely in their amino acid sequences among the cysteines [65]. Unlike the MTs of most other animal species, the snail MTs present homometallic stoichiometry in *in vivo* conditions [67,68], making these proteins interesting molecular models for studying metal-protein interactions.

**Figure 6:**
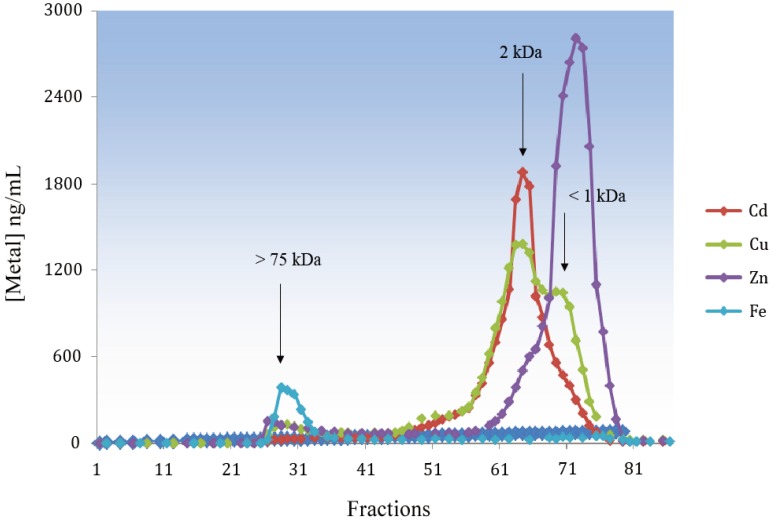
Cd, Cu, Zn and Fe profiles from *Octopus* liver cytosol after gel filtration chromatography on Sephadex G-75.

Cephalopods are considered the most intelligent and most complex invertebrates with a well-developed brain and a widespread presence in every marine environment [69]. The presence of MT-like proteins has been reported for *Todarodes pacificus* [70] and *Octopus vulgaris* [71,72]. In *Octopus* fed with mussel previously exposed to waterborne Cd, we found that most of the Cd in cytosolic liver extracts was bound to ligands with a molecular weight lower than that of classic MTs (Figure 6), and estimated to be between two and three kDa. This peak was also rich in Cu and contained a lower percentage of Zn. We assume that MT could be cleaved in small peptides due to the presence of highly active proteases that we failed to inhibit; the presence of a single domain peptide cannot be excluded. Data on cephalopod MTs are controversial. Craig *et al.* [73] concluded that MTs are probably not involved to a significant extent in metal binding in the squid, *Loligo forbesi*, whereas Raimundo *et al.* [72] claimed that the high levels of Zn, Cu and Cd in the digestive gland of *O. vulgaris* must be ascribed to the presence of MT as suggested by differential pulse polarography determination of the protein, but in this case no molecular weight could be extrapolated.

### 5.2. Deuterostomia

Deuterostomia are a large and heterogeneous group of animals belonging to the Bilateria (Figure 2), in which, unlike Protostomia, the blastopore becomes the anus. Most of the species familiar to common human experience belong to Deuterostomia, including mammals, which have also been investigated in depth for MTs.

#### 5.2.1. Echinodermata

Echinodermata are an invertebrate deuterostome phylum, evolutively close to the Chordata. Two MT isoforms (SpMTA and SpMTB) have been reported in the sea urchin *Strongylocentrotus purpuratus*. These proteins contain 20 cysteines at conserved positions, but show different lengths, SpMTA is 64 amino acids long, while the SpMTB sequence is two amino acids longer [74]. In this species, SpMTA shows a unique molecular structure characterized by the inversion of α and β domains with respect to mammalian MTs. The comparison of sea urchin MTA with mammalian MT-2 showed a two-domain structure in both species, with one domain containing a three-metal cluster and the other a four-metal cluster. However, in mammalian MT-2, the three-metal-containing β-domain corresponds to the *N*-terminal of the sequence and the α-domain is located in the *C*-terminal part, whereas in sea urchin MTA the *N*-terminal α-domain contains four metal ions and the *C*-terminal β-domain three metal ions [75].

#### 5.2.2. Chordata

Compared with the complexity of MT evolution in invertebrates, the differentiation of the MT family in vertebrates seems more straightforward (Table 1). Most of what we know regards mammalian MTs followed by piscine, avian and more recently reptilian MTs. The high polymorphism of mammalian MTs is not found in the other classes of vertebrates, which present only one or two isoforms. Recent sequence studies revealed that vertebrate MTs are grouped in two major clades: one including fish MTs and the other MTs from all the tetrapods [76,77]. In turn, the second group can be further divided into two: one including MT1 of birds and MT-4 of mammals, and the other comprising all the remaining MTs. Vertebrate MT isoforms present a typical length within the different classes, with two exceptions: mammalian MT-3 and MT-4 and amphibians MTs, which are highly variable.

##### 5.2.2.1. Fish

Most of the sequenced MTs in fish have 60 amino acids and present Cys-X-Cys and Cys-Cys at conserved positions. One of the first studied piscine MTs was from plaice, *Pleuronectes platessa* [78], while the first evidence of two distinct genes, MT-A and MT-B, was reported in trout by Bonham *et al.* [79]. Interestingly, MT-A is preferentially induced by Cd, whereas MT-B represents the constitutive isoform, also being induced by this metal [80,81]. Based on ion exchange chromatography and amino acid composition, two isoforms, called MT1 and MT2, differing for the presence of an additional glutamic acid, have also been identified in *Carassius auratus.* Both isoforms presented a biphasic CD spectrum as a direct consequence of the cadmium-thiolate cluster structure common to this protein [82]. Recently, mud loach (*Misgurnus mizolepis*) was also shown to have two different isoforms sharing a high degree of homology in amino acid and nucleotide sequences [83]. One of the proposed functions of MT is linked to its antioxidant activity [84,85] and we also tested this role in fish. Epithelial cells from a carp cell line exposed to a variety of oxidative stressors were protected by MT pre-induction by Cd-exposure, demonstrating the involvement of MT in radical scavenging [86]. Lipid peroxidation, GSH concentration and antioxidant enzyme activities were little affected in *S. aurata* exposed to waterborne Cd, and the negative effects of Cd accumulation in tissues were probably counteracted by MT induction [87]. Fish are commonly used as indicators of environmental chemical pollution, and MT induction is considered a useful biomarker of exposure to trace metals, namely Cd, Cu and Hg. Fish are poikilothermic animals, whose biochemical parameters are influenced by temperature. Accordingly, higher levels of MT were measured in the liver of goldfish injected with Cd at 20 °C compared to those injected at 10 °C [88]. Tom *et al.* [89] demonstrated that MT-mRNA detected in *Lithognathus mormyrus* by a cDNA probe can be considered a useful tool for monitoring heavy metals in the marine benthic habitat of the Mediterranean Sea showing that MT-mRNA levels were reduced with the increasing distance from the pollution source of the Kishton River opening. Using the same cDNA probe in *S. aurata*, Cu exposure was demonstrated to cause an increase in MT-mRNA and CuMT. Both these parameters proved useful biomarkers of an early cellular response to environmental metal exposure [58,90].

##### 5.2.2.2. Amphibians

In spite of their importance as pets, food, a source of bioactive molecules and animal models, e.g., *Xenopus*, only five complete sequences and one partial sequence are present in GenBank, and the first comparative analysis on the primary structure was reported by Trinchella *et al.* [77] in 2012. No MT isoforms have been found in the amphibians investigated so far, though the paucity of data precludes any conclusion on the entire class. Notwithstanding the presence of only one isoform, an unexpected variability was found in: (a) the protein length, which can vary from 60 amino acids in *Ambystoma mexicanus* to 62 and 63 amino acids in *Rana esculenta* and *Triturus carniflex* respectively; (b) the primary structure, e.g., the presence of histidine in *Xenopus*; and (c) the length in 3' untranslated regions of the MT gene, which in *Xenopus* reaches a maximum of 500 nucleotides. The latter could affect the stability of the MT transcript or susceptibility to different regulatory mechanisms [77].

##### 5.2.2.3. Reptiles

Reptile MTs had been scarcely investigated until Trinchella *et al.* [76] cloned and sequenced MTs in squamates. The sequences obtained showed that MTs in these reptiles contain 63 amino acids, 20 of them cysteines. Some species present a *C*-terminal histidine, typical of the avian MT2, which in effect clusters together with these reptilian isoforms in the phylogenetic tree obtained with the MT amino acid sequences [76]. Structural studies suggested this *C*-terminal histidine may be involved in metal binding and in the formation of a bridge between two MTs with consequent dimerization [91]. One important characteristic of reptilian MTs is the lack of polymorphism; only one isoform was expressed in all the reptiles studied so far. Our comparative research on MTs also addressed *Caretta caretta* and *Chelonia mydas*: significantly higher levels of hepatic Cu-MT were found in *C. mydas* paralleling Cu levels in the tissue due to the foraging habits of this herbivorous species [92].

##### 5.2.2.4. Birds

The first complete study on MT characterization in avian species was reported by Weser *et al.* [93], while the first avian MT sequence was obtained in 1988 by McCormick *et al.* [94] from chicken liver. MT was subsequently isolated and characterized also in quail [95]. At least 14 sequences are currently present in GenBank, including MTs from both captive and wild birds, e.g., chicken (*Gallus gallus*), mallard (*Anas platyrhynchos*), cormorant (*Phalocrocorax carbo*). In birds, at least two genes, MT1 and MT2, are present coding for two divergent isoforms of 63 residues, including 20 cysteines at conserved positions [96,97]. The *C*-terminal histidine seems to be characteristic of the MT2 isoform, while MT1 presents a lysine. The simple pattern of avian MT isoforms was also confirmed in woodcock (*Scolopax rusticola*): after ion-exchange chromatography one main isoform was prominently expressed in the kidney [9]. Birds present a wide variety of adaptations to flight, ranging from the pheasant, which has a white pectoral muscle, to migrating birds like ducks and woodcocks, which have a red pectoral muscle. In the latter case, the strong aerobic metabolism of the red muscle fibers will generate oxidative stress and it would be interesting to investigate whether MTs, as free radical scavengers, are differentially expressed in the muscle of migrating birds.

##### 5.2.2.5. Mammals

**Figure 7:**
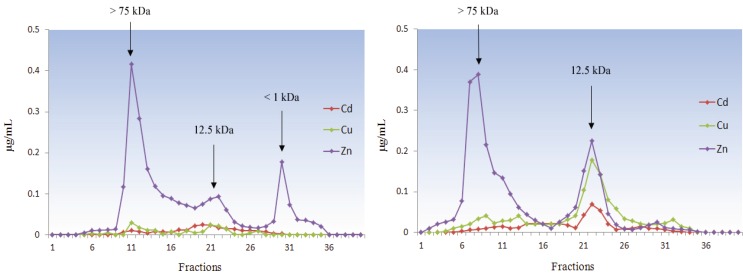
Metal profiles from kidney cytosols of young (**left**) and adult (**right**) *Tursiops truncatus* after gel filtration chromatography.

It is no coincidence that MT was first discovered in the kidney cortex of horse and then extensively studied in primates and rodents. As a mammal, *Homo sapiens* has focused research on itself and on its related species, namely the mammalian animal models and domestic animals. Therefore, most available data on MTs are related to these few species that are beyond the scope of our review and they will only be reported for comparison. Four major isoforms (MT-1 to MT-4) have been identified in mammals and are the paradigm of MT structure and function as reported in Section 3 and Section 4 of this paper. The phylogenetic analyses show that all four major genes originated through a single duplication event prior to the radiation of mammals and the MT-1 gene expanded further in the primate lineage reaching in humans a total of 13 paralogs, five of which are pseudogenes [98]. MTs have been poorly investigated in a very common species, the wild boar (*Sus scrofa*), and in the related domestic pig *Sus scrofa domestica* [99]. By contrast, animals far from ordinary life but much beloved like marine mammals have been investigated for MTs [100,101]. In the harbor porpoise (*Phocoena phocoena*), a small cetacean living in northern Europe waters, MT has been isolated from liver and kidney, confirming a key role for this protein in Zn and Cu homeostasis and in Cd detoxification also in this species [102]. Metal and MT concentrations in tissues can be influenced by several exogenous and endogenous factors, such as environmental conditions, diet composition, gender and age [9,92,103]. In bottlenose dolphins (*Tursiops truncatus*) from the Mediterranean Sea we found that age is a prominent factor affecting kidney MT concentration and the metals bound to the protein (Figure 7).

Diet also influenced the MT concentration and metal speciation in two unconventional mammals from the Quaderna Valley, an unpolluted area not far from the city of Bologna (Italy) that we have recently studied [104]: in the kidney of badger (*Meles meles*), an omnivore that preferentially feeds on earthworms, we isolated MT binding Zn, Cu and Cd in equal amounts, while MT was present only in traces in the kidney of the strictly carnivorous red fox (*Vulpes vulpes*), in spite of the apical position of this species in the trophic chain. It is noteworthy that *M. meles* and *S. rusticola* two species of vertebrates not phylogenetically close but sharing a diet based on earthworms present similar MT chromatographic patterns [9,105]. Other well-known unconventional mammals are small wild rodents, in particular the bank vole *Myodes glareolus,* the western Mediterranean mouse *Mus spretus* and different species of the *Microtus* genus, that have been sampled in biomonitoring studies using MTs as a biomarker of environmental heavy metal exposure [103,105]. Among these mammals, the free-living mouse *M. spretus* represents a promising option to conventional animal models due to its high genetic homology with the laboratory mouse *Mus musculus*. In this case, the huge body of knowledge acquired on *M. musculus* can be successfully applied to *M. spretus* as reported by Garcìa-Sevillano *et al.* [106], confirming the possibility of applying metallomic techniques in environmental pollution assessment using *M. spretus* as a bioindicator.

## 6. Conclusions

**Figure 8:**
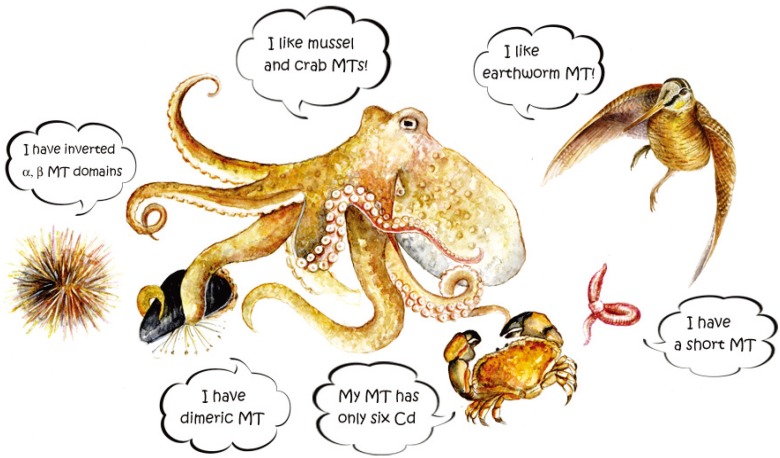
The “First MT meeting on Unconventional MTs” organized by unconventional animals is reported in this cartoon. Watercolor by Gloria Isani.

Our review focused mainly on the variety of MT isoforms in different species of unconventional animals. The complexity of the isoform patterns of each species does not seem to follow an evolutionary trend, *i.e.*, becoming increasingly complex from the least evolved invertebrates to the most evolved vertebrates. Because there is no apparent link with diet or environment, MT isoforms and their molecular characteristics seem to be controlled simply by a stochastic principle. Only in these organisms and mostly in invertebrates it is possible to find unconventional MTs, which seem distantly related to each other. Key examples of this heterogeneity include: an unusual short isoform of 41 amino acids in the earthworm *E. fetida*; MT structured in two β-clusters binding only six bivalent metal ions in the mangrove crab *S. serrata*; highly polymorphic MTs in the mussel *M. edulis*, 71–72 amino acids long and containing 23 and 21 cysteine residues respectively, which could originate intermolecular bridges; a Cd-MT gene in the Roman snail *H. pomatia* encoding a specific isoform; the sea urchin *S. purpuratus* showing a unique MT molecular structure with the inversion of α and β domains. These comparative MT studies have disclosed molecular “oddities” that together could shed more light on the true function of MTs (Figure 8). According to Blindauer and Leszczyszyn [14], the reason why the true function of MTs is still a matter of debate is that these “elusive” proteins simply do not have a unified role, but we do not exclude that the true function has not yet been discovered.
